# 

**Published:** 1818-10

**Authors:** 


### BOOKS ANNOUNCED FOR SPEEDY PUBLICATION.

The third edition is in the press of Pharmacologia; or the History of Medicinal Substances, especially as it regards their Chemical Habitudes and Relations; with a View to render the Art of Prescribing and of Composing Extemporaneous Formulæ less empirical and more successful, by the establishing it upon fixed and scientific Principles; illustrated by Formulæ, &c. By JOHN AYRTON PARIS, M.D. F.L.S. &c.

Pathological and Surgical Observations on Diseases of the Joints. By B. C. BRODIE, F.R.S. &c. Illustrated by plates: is in the press.

Shortly will be published, a Practical Treatise on Tropical Dysentery, more particularly as it occurs in the East and West Indies; illustrated by Cases and Dissections. By R. W. BAMFIELD, late Surgeon of the Belliqueux and Warrier.

### LECTURES ABOUT TO BE DELIVERED IN LONDON.

*Medical School of St. Thomas's and Guy's Hospitals.*—The usual Lectures at these adjoining Hospitals will be given as follows:—At St. Thomas's, Anatomy and the Operations of Surgery, by Mr. Astley Cooper and Mr. Henry Cline; Principles and Practice of Surgery, by Mr. Astley Cooper.—And the Winter Course of Lectures will commence at Guy's the 2d of October, viz. Practice of Medicine, by Dr. Currey and Dr. Cholmeley; Chemistry, by Dr. Marcet; Experimental Philosophy, by Dr. Evans; Theory of Medicine and Materia Medica, by Dr. Curry and Dr. Cholmeley; Midwifery and Diseases of Women and Children, by Dr. Haighton; Physiology, or Laws of the Animal Economy, by Dr. Haighton and Dr. Blundell; Structure and Diseases of the Teeth, by Mr. Bell.—[*Adv.*]

*Theatre of Midwifery*, 68, *Berner's-street.*—Dr. Clough will commence his Autumnal Course of Lectures on the above Science, on Monday morning the 5th of October, at half-past ten, and an evening Course at seven.—[*Adv.*]

Dr. Ramsbotham will begin his Lectures on the Science and Practice of Midwifery and the Diseases of Women and Children, on Monday, October 5th, at 11.

Dr. Clutterbuck will begin his Autumn Course of Lectures on the Theory and Practice of Physic, Materia Medica, and Chemistry, on Friday, Oct. 2, 1818.

Dr. M B. Davis will commence his next Course of Lectures on that Branch of the Practice of Medicine which relates to the Diseases and Medicinal Management of Children and Young Persons, early in the ensuing month.

Dr. Davis will commence his Winter Lectures on the Theory and Practice of Midwifery, and on the Diseases of Women and Children, on the 5th of October.

Dr. George Gregory and Dr. Clowes will begin their first Course of Lectures on the Theory and Practice of Physic, on Wednesday, October 7th.

Mr. Taunton's Winter Course of Lectures on Anatomy, Physiology, Pathology, and Surgery, will commence on Saturday, October 3d.

J. C. Carpue, F.R.S. will commence his Anatomical Lectures on Thursday, the first of October.

Mr. Guthrie, Deputy-Inspector of Military Hospitals, will commence his Winter Course of Lectures on Surgery, on Monday, the 5th of October.

The Lectures on Anatomy and Surgery, in the School of Great Windmill-street, begin on the first of October.

Mr. Mackenzie's Winter Course of Lectures, on the Diseases of the Eye, will commence on the 5th of October.

---

### CATALOGUE OF MEDICAL BOOKS.

Observations on the Symptoms and Specific Distinctions of Venereal Disease. By Richard Carmichael, M.R.I.A. &c. &c. 8vo. 9s.

Medical and Chemical Remarks on Arsenic; to which are added, Cases of Recovery. By John Marshall. 8vo. 6s.

An Inquiry into the Influence of Situation in Pulmonary Consumption, and on the duration of Life. By J. G. Mansford. 8vo. 5s.

Observations on the different Kinds of Small-Pox, and especially on that which sometimes follows Vaccination. By Alexander Monro, M.D. F.R.S.E. 8vo. 10s. 6d.

Directions for the Treatment of Persons who have taken Poison, and those in a State of Apparent Death; with an Appendix on suspended Animation and the Means of Prevention. Translated from the French by R. H. Black, Surgeon. 12mo. 5s. boards.

A Manual of Practical Anatomy for the use of Students engaged in Dissections. By Edward Stanley, Esq. Demonstrator of Anatomy at St. Bartholomew's Hospital. 12mo.

The London Dispensatory; containing the Elements of Pharmacy, the Botanical Description, Natural History, Chemical Analysis, and Medical Properties, of the Substances of the Materia Medica; the Pharmaceutical Preparations and Compositions of the Pharmacopœia; forming a Practical Synopsis of Materia Medica, Pharmacy, and Therapeutics. By A. T. Thompson. 8vo. 16s.

A Catalogue of Books in Anatomy, Medicine, Surgery, Midwifery, &c. &c.; with a Table of Contents, methodically arranged, including Books in every Department of Literature; as sold by Burgess and Hill, at their Library, 55, Great Windmill-street, Haymarket.

---

### *TO SUBSCRIBERS.*

*The late addition to the Editorial Department, and the introduction of a new feature into the character of this Journal, furnish the Proprietors with an occasion for addressing their Subscribers, which they embrace with no small degree of satisfaction; they may indeed be allowed to say, not without feelings of pride, from reflecting on the exertions they have made to render the Work worthy of the attention of the Medical Profession.*

*The very flattering reception it has for many years experienced, not only in England, but in all parts of the world in which medical science is liberally cultivated, proves their exertions have not been made in vain.*

*Anxious to embrace every mean which may contribute more perfectly to fulfil their professed intentions, they have lately seized an opportunity to engage the services of two additional Editors, whose talents and zeal for the improvement of Medical Science cannot fail to add considerably to the value of the work to which they are devoted. The arrangements they have made for regular correspondence with men of the most distinguished character in the profession, on the continents of Europe and America, furnish them, also, with the earliest information of all the important foreign discoveries and improvements in Medicine, Surgery, and their auxiliary branches of Science; the application of which acquirements will form a new Department in this Journal, which must prove highly interesting to their readers. The present Number, they trust, will be considered valuable in this respect, but they do not adduce it as a specimen of what may be effected shortly from this arrangement.*

*It must be evident, that no expense has been withheld by the Proprietors that might contribute to render the work worthy of universal attention by the Professors of Medicine; the gratification felt from reflecting on the high reputation it has possessed for a long series of years, notwithstanding the endeavours of its rivals, renders pecuniary considerations of trifling import, and imparts a degree of energy to their exertions and those of the Editors, which may add to the powers, even of those the most ardently devoted to the improvement of science.*

---

*The favours of* Dr. Albers, *of Bremen, with those of several other Correspondents, shall be noticed in our next.*

